# Adjudicating the Diagnosis of Immune Thrombocytopenia in a Clinical Research Study

**DOI:** 10.1055/a-2054-3923

**Published:** 2023-05-03

**Authors:** Caroline Gabe, Syed Mahamad, Melanie St John, Joanne Duncan, John G. Kelton, Donald M. Arnold

**Affiliations:** 1 Michael G. DeGroote Centre for Transfusion Research, Department of Medicine, McMaster University, Hamilton, Ontario, Canada; 2 Department of Medicine, McMaster University, Hamilton, Ontario, Canada

Immune thrombocytopenia (ITP) is a common autoimmune bleeding disorder. Establishing the diagnosis of ITP is challenging due to the lack of a reliable biomarker and nonspecific clinical criteria. 1 2 A diagnosis of exclusion, ITP can only be established once other thrombocytopenic syndromes have been excluded. As a result, patients presenting with thrombocytopenia are often misdiagnosed as having or not having ITP, which can lead to delays in proper care and inappropriate use of immune-suppressant therapies. 3 To improve diagnostic accuracy, we implemented an adjudication process for the diagnosis of thrombocytopenic disorders in a clinical registry based on previous clinical studies 4 and consensus statements. 5 The objective of this study was to describe the methods and process of adjudicating the diagnosis of thrombocytopenia in a research context.

Consecutive adults with thrombocytopenia who were referred to the McMaster ITP Registry were included. The registry is a single-center prospective, longitudinal study of consecutive adult patients presenting for investigation of thrombocytopenia to the tertiary hematology clinic at McMaster University in Canada. We included patients enrolled in the registry from inception (January 2010) until December 2019. All patients had platelets less than 150 × 10 ^9^ /L and no exclusions were applied.

The cause of the thrombocytopenia was assessed by two hematologists with expertise in ITP who worked in the clinic. Patients were followed prospectively every 6 months until discharge or death. Baseline blood samples were collected for laboratory evaluations and repeated as necessary, including complete blood counts which were done at every visit. Bleeding assessments were done by a dedicated research assistant at each visit using the ITP Bleeding Scale. 6 The diagnosis was assessed at each visit based on corollary investigations (e.g., liver enzymes indicative of liver disease, cytopenias, or bone marrow examination results indicative of myelodysplasia, etc.). When there was insufficient information and a working diagnosis could not be assigned, patients were classified as “no clear cause of thrombocytopenia identified”. The cause of the thrombocytopenia was adjudicated when: (1) no clear cause of thrombocytopenia was identified; (2) the diagnosis changed from one visit to another; or (3) the thrombocytopenia occurred in the context of pregnancy.

**Fig. 1:**
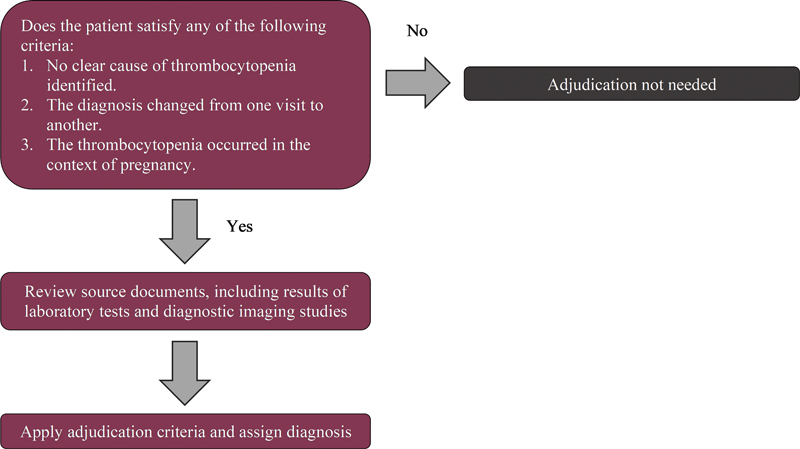
Flowchart demonstrating the application of the adjudication process for patients with thrombocytopenia in a research context.

Adjudication was done by an independent hematologist and a research associate ( Fig. 1 ). All source documents were reviewed including results of laboratory tests and diagnostic imaging studies. A platelet count rise following high-dose intravenous immune globulin or high-dose corticosteroids was used to classify patients as having ITP, which aligns with a previous agreement study 4 and consensus statement. 5 The adjudication criteria, which were refined iteratively and finalized during the study, are shown in Table 1 . The adjudication process was developed for use in a research context.

**Table 1:** Adjudication criteria for the diagnosis of thrombocytopenia

Diagnosis	Adjudication criteria
Mild thrombocytopenia	• Platelet count consistently between 100–150 × 10 ^9^ /L. For pregnant patients, consider gestational thrombocytopenia. Mild thrombocytopenia supersedes other diagnoses (e.g., hepatitis C or family history of thrombocytopenia)
Primary ITP	• Patients with platelets < 100 × 10 ^9^ /L and no other diagnosis; and a platelet count response a to corticosteroid or high-dose IVIG. If platelet counts improve > 100, the diagnosis should remain primary ITP
Secondary ITP • Antiphospholipid syndrome • Chronic lymphocytic leukemia • Common variable immune deficiency • Evan's syndrome • *Helicobacter pylori* ( *H. pylori* ) • Hepatitis C • HIV • Non-specific infection • Pregnancy-associated ITP • Lymphoma • Systemic lupus erythematosus • Sarcoidosis • Other autoimmune disease	• Once a diagnosis of secondary ITP is made it should remain as such even if the underlying disorder is treated and the ITP persists • For pregnancy-associated ITP platelets should improve with ITP treatment; if the ITP persists postpartum or predates pregnancy, the diagnosis should be primary ITP • For *H. pylori* -associated ITP, there should be evidence of *H. pylori* eradication and improvement in platelet count (definite); or evidence of active *H. pylori* infection and improvement of platelet count with treatment, even without evidence of eradication (probable)
Drug-induced ITP	• Onset of thrombocytopenia is typically 5–10 days after initial drug exposure and platelet count recovery typically occurs after discontinuing the drug, with no other drugs implicated. Confirmation requires either a drug challenge or the demonstration of drug-induced platelet antibodies.
Non-immune thrombocytopenia • Alcohol related • Familial • Incidental thrombocytopenia in pregnancy (gestational thrombocytopenia) • Hypertensive disorders of pregnancy • Liver disease • Splenomegaly/hypersplenism • Myelodysplastic syndrome • Pseudothrombocytopenia • Drug-induced bone marrow suppression • Thrombocytopenia associated with malignancy including aplastic anemia	• For familial thrombocytopenia, platelet count should be below 100 × 10 ^9^ /L (otherwise, classify as mild thrombocytopenia), with a family history in first-degree relatives • For incidental thrombocytopenia of pregnancy (gestational thrombocytopenia), platelet count is typically above 70 × 10 ^9^ /L during pregnancy, normalization of platelet count postdelivery, no history of thrombocytopenia (except during a prior pregnancy), and no thrombocytopenia in the fetus or newborn • Fatty liver disease alone (without other stigmas of chronic liver disease) is typically not a cause of non-immune thrombocytopenia • For patients with spleen enlargement, classify as splenomegaly unless the patient has liver cirrhosis or portal hypertension, in which case classify as liver disease • The diagnosis of myelodysplastic syndrome can be presumed even without bone marrow evaluation when other features are present (e.g., variable sized platelets, hypogranular platelets, and hypolobulated neutrophils on the peripheral blood smear) • For pseudothrombocytopenia, platelet clumping observed in the peripheral blood smear with a routine complete blood count, and the platelet count normalizes when citrate or heparin is used in the blood collection tube
Other thrombocytopenia disorders • Cyclical thrombocytopenia • Heparin-induced thrombocytopenia • Thrombotic microangiopathies	• For cyclical thrombocytopenia, there should be evidence of large platelet count fluctuations independent of treatment. If fluctuations resolve but platelet count stays low, the diagnosis of ITP should be considered
Unknown	• Does not meet criteria for any category, or meets criteria for more than one category, or data are not available

Abbreviations: HIV, human immunodeficiency virus; ITP, immune thrombocytopenia; IVIG, intravenous immune globulin.

a Response is defined as an increase in baseline platelet count to 50 × 10 ^9^ /L or higher and doubling of baseline platelet count within 4 weeks.

Up to December 2019, 789 patients with complete data were enrolled in the McMaster ITP Registry; of those, 157 (19.9%) met criteria for adjudication. After initial review, 27 patients were considered classification errors; thus, the remaining 130 patients ( *n* = 195 study visits) with thrombocytopenia constituted this adjudication study population ( Table 2 ). The mean (range) age was 62.3 years (22–93 years) and 71 were female (54.6%). The criteria for adjudication were: a change in the diagnosis from one visit to the next ( *n* = 77; 59.3%), no clear cause of the thrombocytopenia was identified ( *n* = 46; 35.4%), and thrombocytopenia occurring in the context of pregnancy ( *n* = 7; 5.4%).

**Table 2:** Initial and final diagnosis after adjudication among patients with thrombocytopenia enrolled in the McMaster Immune Thrombocytopenia (ITP) Registry

Initial diagnosis (pre-adjudication)	Final diagnosis (post-adjudication)		*N*
Primary ITP	Secondary ITP		10
		APS	2
		Evan's syndrome	2
		Pregnancy related	2
		Rheumatoid arthritis	2
		Hepatitis C	1
		Nonspecific viral illness	1
	Non-immune thrombocytopenia		9
		Gestational thrombocytopenia	2
		MDS	2
		Pseudothrombocytopenia	2
		Splenomegaly	2
		Liver disease	1
	Mild thrombocytopenia		5
	Unknown		2
Secondary ITP	Change in underlying cause of secondary ITP		2
		Hepatitis C	1
		*Helicobacter pylori*	1
	Primary ITP		3
	Mild thrombocytopenia		1
Non-immune thrombocytopenia	Change in underlying cause of non-immune thrombocytopenia		10
		Liver disease	5
		MDS	2
		Drug-related bone marrow suppression	1
		Pancytopenia	1
		Splenomegaly	1
	Primary ITP		7
	Secondary ITP		1
		Pregnancy related	1
	Unknown		1
	Mild thrombocytopenia		1
Unknown	Primary ITP		15
	Secondary ITP		1
		Hepatitis C	1
	Non-immune thrombocytopenia		10
		Liver disease	2
		Splenomegaly	2
		Alcohol related	1
		Drug-related bone marrow suppression	1
		Familial thrombocytopenia	1
		Hypersplenism	1
		Pancytopenia	1
		Pseudothrombocytopenia	1
	Other thrombocytopenia disorder		2
Other thrombocytopenia disorders	Mild thrombocytopenia		2
	Non-immune thrombocytopenia		1
		Liver disease	1
Mild thrombocytopenia	Primary ITP		4
	Non-immune thrombocytopenia		4
		Splenomegaly	3
		MDS	1
	Unknown		1

Abbreviations: APS, antiphospholipid syndrome; ITP, immune thrombocytopenia; MDS, myelodysplastic syndrome.

**Fig. 2:**
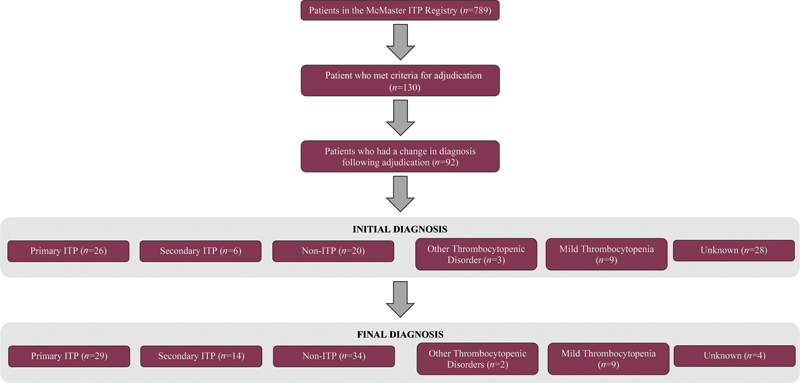
Flowchart showing the number of patients whose diagnosis changed after adjudication.

After adjudication, the diagnosis changed for 92 patients (70.8%; *n* = 150 clinic visits) ( Fig. 2 ). Of those, the diagnosis changed once for 62 (67.8%) patients, and more than once for 30 (32.6%) patients. For patients initially classified as primary ITP whose diagnosis changed after adjudication ( *n* = 26), 10 (38.5%) changed to secondary ITP; 9 (34.6%) changed to non-ITP; 2 (7.7%) changed to unknown diagnosis; and 5 (19.2%) changed to mild thrombocytopenia only. For patients with secondary ITP whose diagnosis changed after adjudication ( *n* = 6), the underlying cause of the ITP changed for 2 patients (33.3%), 3 patients (50.0%) changed to primary ITP, and 1 (16.7%) changed to mild thrombocytopenia only. For patients with non-ITP whose diagnosis changed ( *n* = 20) after adjudication, the underlying cause of the non-ITP changed for 10 patients (50.0%), 7 patients changed to primary ITP (35.0%), 1 (5%) changed to secondary ITP, 1 (5.0%) changed to unknown, and 1 (5.0%) changed to mild thrombocytopenia. For patients whose diagnosis was unknown initially ( *n* = 28), 15 (53.6%) patients were resolved to primary ITP, 1 (3.6%) was resolved to secondary ITP, 10 (35.7%) were resolved to non-ITP, and 2 (7.1%) were changed to “other” (e.g., a different diagnosis that does not fit the preestablished criteria) after adjudication. For patients with an initial diagnosis of “other” ( *n* = 3), 2 (66.7%) changed to mild thrombocytopenia only and 1 (33.3%) changed to non-ITP after adjudication. For patients with an initial diagnosis of mild thrombocytopenia ( *n* = 9), 4 (44.4%) changed to primary ITP, 1 (11.1%) to unknown, and 4 (44.4%) to non-ITP after adjudication.

The process for establishing the clinical diagnosis of thrombocytopenic disorders is not standardized, prone to ascertainment bias, and influenced by provider and region. To improve diagnostic accuracy, we developed an adjudication process for patients with thrombocytopenic disorders. The adjudication criteria were applied to patients whose diagnosis changed from one visit to the next, who had thrombocytopenia in the context of pregnancy (because of overlap with other causes during pregnancy), and when no cause of the thrombocytopenia was identified. The adjudication criteria included hierarchical rules to determine when a patient should be classified as primary ITP, secondary ITP, non-ITP, or when the diagnosis was uncertain and described as “unknown.” Our results showed that adjudication led to a change in the diagnosis for 70.7% of patients who met the adjudication criteria (92/130), and that the five most common changes were from unknown to primary ITP ( *n* = 15); primary ITP to secondary ITP ( *n* = 10); unknown to non-ITP ( *n* = 10); different cause for non-ITP ( *n* = 10); and primary ITP to non-ITP ( *n* = 9).

Strengths of this study were the application of a systematic approach to the diagnosis of thrombocytopenic disorders, the variety of thrombocytopenic conditions included, and the use of independent adjudicators. Limitations were the time-intensive nature of the process which may make it difficult to apply in a busy clinical practice, and the need for future prospective validation studies.

Adjudication, or “to act as a judge,” is a process by which expert guidance is used to classify outcomes. 7 It is commonly used in clinical trials when the outcome of interest does not have an established or standard definition and outcome assessment requires the synthesis of data elements and assessor judgment. The adjudication process can improve reliability and avoid misdiagnosis by reducing random and systematic error in classification procedures. 8 In hematology clinical trials, adjudication has been used for the assessment of deep vein thrombosis, 9 postthrombotic syndrome, 10 and bleeding outcomes. 8 The diagnosis of ITP is another outcome that is subject to ascertainment bias and thus well-suited for an adjudication process.

In conclusion, we described the process and impact of adjudication on the diagnosis of thrombocytopenic disorders. Adjudication in this context can improve the accuracy of patient classification and avoid case mix in clinical trials.
